# Primary gastrointestinal stromal tumor of the left hepatic lobe: a case report and a review of the literature

**DOI:** 10.1093/jscr/rjab572

**Published:** 2021-12-23

**Authors:** Athanasios Piachas, Andreas Tooulias, Georgios Tsoulfas

**Affiliations:** Division of Transplant Surgery, Aristotle University of Thessaloniki, Ippokratio General Hospital, Thessaloniki, Greece; Department of Surgery, Aristotle University of Thessaloniki, Papageorgiou General Hospital, Thessaloniki, Greece; Division of Transplant Surgery, Aristotle University of Thessaloniki, Ippokratio General Hospital, Thessaloniki, Greece

## Abstract

Gastrointestinal stromal tumors (GISTs) can arise from any site of the gastrointestinal tract. These tumors are known to originate from the interstitial cells of Cajal, located in the gastrointestinal mesenchyme. In the case presented, a 37-year-old Caucasian male was admitted to our Surgery department with 2-month history of mild abdominal pain, early satiety and flatulence. The computed tomography revealed a huge mass in the left hepatic lobe consisting of both spindle and epithelial cells. Immunohistochemistry revealed strong CD117 positivity expression. Only a few other cases of liver GIST have been reported in the literature.

## INTRODUCTION

Gastrointestinal stromal tumors (GISTs) are quiet rare tumors, with an estimated annual incidence of 10–20 per million. They are usually found in the gastrointestinal tract, with the stomach and small intestine being the most frequent site of appearance (>90%) [1–3]. Αpproximately, 20–30% of the cases are already metastatic upon first diagnosis [4]. The majority of patients are between 55 and 65 years old [1]. These tumors rarely appear to arise in locations outside the gastrointestinal tract, such as the liver, the gallbladder, the pancreas and the omentum [5–9]. These cases are described as extra-GISTs (EGISTs).

Studies have shown that these tumors have the same phenotypic characteristics as the interstitial cells of Cajal (ICC). ICCs constitute a composite network of cells located in the gastrointestinal tract wall, known as the pacemakers of the gut, and they were found to express the kit protooncogene. The product of this gene’s expression is transmembrane tyrosine-kinase receptor (CD117) [10, 11].

## CASE PRESENTATION

A 37-year-old Caucasian man was admitted to our Surgery department with a 2-month history of mild abdominal pain, early satiety and flatulence. On physical examination, distention of the abdomen was obvious and a palpable mass was readily palpated.

**Figure 1:**
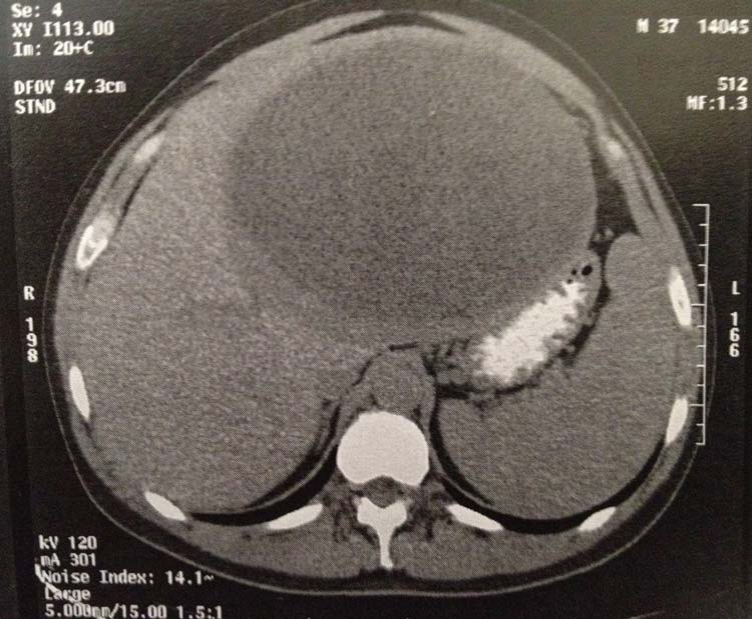
Computer tomography image showing a 23 × 22 × 16-cm lesion arising from the left lobe of the liver.

The computed tomography (CT) scan revealed a semi-solid mass in the left liver lobe, measuring 22 × 21 × 16 cm with peripheral enhancement after contrast administration (Fig. 1). An upper and lower endoscopy revealed no other lesions of the gastrointestinal tract. Tumor markers, such as a-phetoprotein (AFP) and carcinoembryonic antigen (CEA) were within the normal limits.

**Figure 2:**
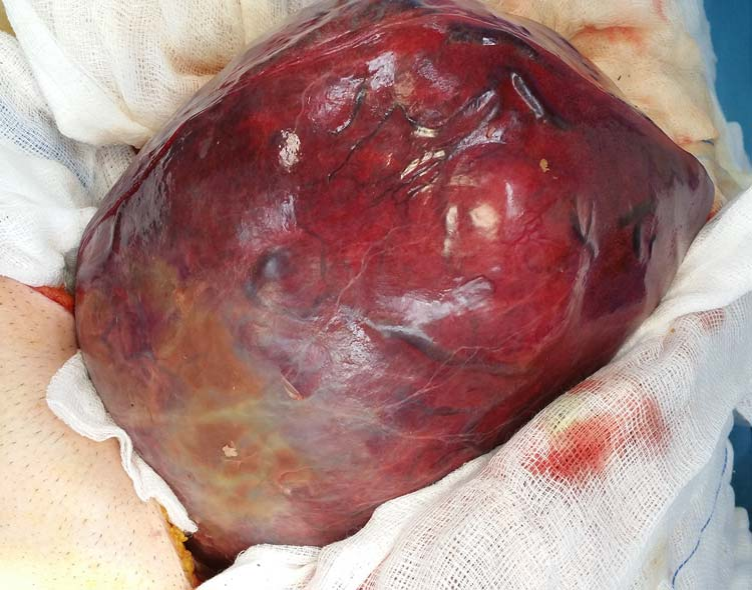
Intraoperative view of the tumor before its resection.

**Figure 3:**
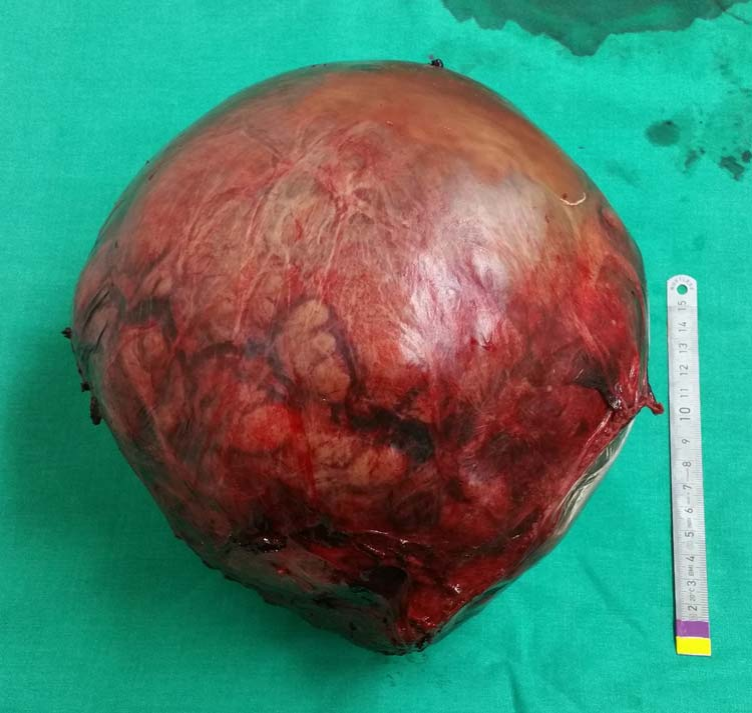
Intraoperative view of the tumor after its resection.

Intraoperatively, the mass was originating from the left hepatic lobe (Fig. 2). A thorough examination of the peritoneal cavity revealed no additional lesions. The patient underwent a left hepatectomy with macroscopic tumor negative margins (Fig. 3). No perioperative incident was reported.

Immunohistochemistry demonstrated strong positivity for the cluster CD117 and vimentin, whereas negativity to the cluster CD34, smooth muscle actin (SMA) and pancreatin. Microscopically, the hepatic tumor consisted of both spindle and epithelial cells with a mitotic count of 20 mitoses per 50 high-power fields. All these pathological findings indicated the presence of a gastrointestinal stromal tumor of the liver.

The patient recovered well and was discharged on the seventh postoperative day.

## DISCUSSION

Gastrointestinal stromal tumors constitute a specific group of tumors located in different sites of the gastrointestinal tract. The most common site of appearance is the stomach (60–70%), followed by the small intestine (20–25%), colon and rectum (5%), as well as the esophagus (<5%) [1, 2]. However, they can also occur in other sites, such as the liver, the gallbladder, the pancreas and the omentum [5–9]. These are characterized as extragastrointestinal stromal tumors—EGISTs and they represent just 1% of all GISTs of defined origin [1]. The clinical appearance is non-specific. General symptoms such as abdominal pain, nausea, flatulence and early satiety can be present as a result of the pressure of the tumor to the adjacent organs; hence the diagnosis is usually belated.

Three criteria must be taken into consideration when classifying a tumor as GIST: (i) the tumor’s location in or adjacent to the gastrointestinal tract, (ii) histologic appearance similar to GIST and (iii) a definite immunoreactivity for CD117 [12]. Particularly, CD117 is a very sensitive marker for the gastrointestinal stromal tumors, since it has been expressed in the majority of these tumors (85%) [13].

GISTs are characterized by a wide range of malignancy risk. Fletcher *et al.* developed the NIH GIST Consensus Criteria, which take into consideration the tumor size and the mitotic count [3]. Joensuu has proposed a modification of the NIH-Fletcher criteria, which, additionally, takes into consideration the presence of tumor rupture. Tumor rupture is an independent prognostic factor over size, site and mitotic count [14].

In our case the radiological findings were primarily suggestive of a cystadenocarcinoma, thus the immunohistochemical findings played a vital role in establishing the correct diagnosis. Only 11 other cases with EGIST of the liver have been reported in the literature [9]. Consequently, a fully preoperative examination, including radiological examination together with an upper and lower endoscopy, is necessary in order to establish the correct diagnosis and organize the right therapeutic plan for the patient.

## CONCLUSION

In conclusion, primary gastrointestinal stromal tumors of the liver are still extremely rare, thus the preoperative diagnosis remains a challenge for the clinician. Findings from the histological and immunohistochemical examination, such as positive staining for CD117 and CD34, are very helpful in the direction of the correct diagnosis. The gold standard treatment for the patients with a gastrointestinal stromal tumor still remains the complete tumor resection with microscopic negative tumor margins.

## AUTHOR’S CONTRIBUTIONS

AP reviewed the literature, wrote the manuscript and edited the images. AT reviewed the literature. GT made a contribution to drafting and reviewed the manuscript.

## CONFLICT OF INTEREST STATEMENT

The authors declare that they have no conflict of interest.
